# Huachansu mediates cell death in non-Hodgkin’s lymphoma by induction of caspase-3 and inhibition of MAP kinase

**DOI:** 10.3892/ijo.2015.3044

**Published:** 2015-06-11

**Authors:** Ekem T. Efuet, Xiao-Ping Ding, Carrie Cartwright, Yong Pan, Lorenzo Cohen, Peiying Yang

**Affiliations:** Department of General Oncology, Integrative Medicine Program, The University of Texas MD Anderson Cancer Center, Houston, TX 77030, USA

**Keywords:** Huachansu, non-Hodgkin’s lymphomas, apoptosis, caspase, MAP kinase

## Abstract

Huachansu (HCS), a hot water extract of the skin glands of *Bufo gargarizans* (*B. melanostictus*), has been used extensively in the treatment of various solid tumors in Asia, particularly in China. However, its effect on the growth of malignancies of hematopoietic origin, particularly lymphomas, is limited. Here we investigated the antiproliferative effect and molecular mechanisms of HCS using non-Hodgkin’s lymphoma (NHL) Raji, Ramos, and Namalwa cells and the mantle cell lymphoma cells SP53. HCS inhibited proliferation in these cell lines with an IC_50_ ranging from 3.1 to 25 μl/ml. At a concentration of 25 μl/ml, HCS triggered a sub-G1 arrest in Ramos cells and induced early to late apoptotic cell death. Cleaved caspase-3 was formed in a concentration-dependent manner in Ramos cells following treatment with HCS for 24 h. Intriguingly, when the Ramos cells were treated with the caspase inhibitor ZDEVD, the apoptotic activity of HCS was partially blocked. Furthermore, HCS also blocked the expression of survivin and pRB proteins in a concentration-dependent manner in Ramos cells. Mechanistically, HCS downregulated both the MAPK gene and proteins in Ramos cells. Collectively, our data suggest that HCS is effective in inducing cell death and apoptosis, in part, by activating caspase-3 activity and suppressing MAP kinase in NHL cells.

## Introduction

Non-Hodgkin’s lymphomas (NHLs) are common hematologic malignancies representing ~5.3% of all cancers in the United States and >50% of all blood cancers (1). B-cell lymphomas are the most common types of NHLs. Despite the considerable research progress on lymphomas, as well as the improved treatment regimens, the survival statistics remain poor, especially for the aggressive forms of NHLs. Currently, combination therapy is the preferred treatment modality for lymphoma, albeit with significant adverse side effects, particularly for the more aggressive types, such as Burkitt’s lymphoma (BL) and mantle cell lymphoma (MCL). The majority of patients with BL respond poorly to the CHOP treatment (2). Similarly, many patients with MCL have a poor response to CHOP, have high rates of relapse, with a median survival rate of 3–5 years (3). Finding drugs that specifically target BL and MCL while sparing normal cells is a major focus of current research. Because treatment failure depends on a complex interplay of factors including tumor biology, pharmacokinetics and pharmacogenomics (4,5), the use of natural products, such as Huachansu (HCS), comprising many bioactive components (6) may prove to be more efficacious than a single agent or single agent combinations.

In traditional Chinese medicine (TCM), the use of Chansu was initially recorded in the Tang Dynasty, >1,000 years ago. Described in the Chinese pharmacopoeia as a detoxicant and an anodyne, its pharmacological attributes include detoxification and resuscitation as well as the ability to reduce swelling and pain associated with infections and malignant cell growth. Its wide use as a local anesthetic, a cardiotonic, and a diuretic has also been recorded. More recently, studies have suggested that Chansu inhibits vasodilation (increased vasoconstriction) and increases vascular resistance and blood pressure via its inherent anti-inflammatory effects and through inhibition of Na^+^, K^+^-ATPase (7). In addition, formulations of Chansu have been used for the treatment of various cancers including hepatic, pancreatic, gastric, lung, skin, and esophageal cancers in oncology clinics in China (8–10). Among the different formulations, Huachansu, an injectable form of Chansu, is one of the most popular formulations and has been extensively used in the treatment of various solid tumors including hepatocellular and non-small cell lung cancer in China (11).

As a form of traditional Chinese medicine approved by the State of Food and Drug Administration (SFDA), HCS and its bioactive components, cardiac glycosides (mainly bufadienolides), exhibited significant inhibitory activity against various human cancer cells, such as human colon cancer cells 26-L5; leukemia cells (K562, U937 and HL-60); hepatocellular carcinoma SMMC-7721, Bel-7402 and HepG2 cells; prostate cancer LNCaP, PC3 and DU145 cells; endometrial (HHUA and HEC-1), and ovarian (SK-OV-3) cancer cells (12–14). Mechanistically, the antiproliferative effect of HCS in the various cancer cells was mediated through cell cycle alteration and induction of apoptosis by modulating apoptosis-related proteins such as Bax, Bcl-2, Fas, Fas-L, survivin, and mitochondria-mediated pathways (15–18), as well as angiogenesis by inhibiting expression of VEGF and EGFR proteins (19,20). Taken together, these studies support the notion that HCS and bufadienolides have the ability to suppress the proliferation of various solid tumors and, possibly, have great potential as anticancer agents. However, the effect of HCS on the growth of malignancies of hematopoietic origin, particularly lymphomas is limited. Case reports from China have demonstrated that HCS alone or in combination with CHOP enhances the response rate of patients with NHLs (21,22) supporting its role in the management of NHLs.

In the present study, we investigated the potential anti-tumor activity and the associated molecular mechanisms of HCS in NHL cells. The antiproliferative effect of HCS and its fraction was evaluated on a number of different NHL cell lines including human Burkitt’s B-cell lymphoma, such as Raji, Ramos, Namalwa and mantle cell lymphoma, SP53 cells. A transcriptome analysis of Ramos cells treated with HCS revealed HCS altered several interesting oncogenes, including MAP kinase. Given that a number of studies have demonstrated that pharmacologically targeting MAPK and inhibiting its activity decrease proliferation in a variety of tumor types (23), including NHL, HCS may have great potential to be developed as a targeted anticancer agent for NHL, particularly B-cell lymphomas.

## Materials and methods

### Chemical, reagents and antibodies

Huachansu was manufactured by Anhui JinChan Biochemistry Sharing Inc. (Anhui, China). DMSO was purchased from Sigma (St. Louis, MO, USA). ZDEVD was purchased from BD Pharmingen (Franklin Lakes, NJ, USA). ZDEVD was dissolved in DMSO and stored at −20°C. In all cases, the final concentration of DMSO was <0.1% (vol/vol). PrestoBlue reagent was purchased from Invitrogen (Frederick, MD, USA). Caspase-3 (E8, sc-7272), caspase-9 (F-7, sc-17784), survivin (D-8, sc-17779), p21 (sc-187), Mcl-1 (sc-12756) were purchased from Santa Cruz (Santa Cruz, CA, USA); anti-cdk2 (BD-610145) and anti-cdk4 (BD-610147) were purchased from BD Transduction Laboratories (Franklin Lakes, NJ, USA); Rb (4H1, CS-9309), phospho-Rb (ser308) (CS-2181), p38MAPK (CS-9212S) were purchased from Cell Signaling (Danvers, MA, USA), pMAPK (V803A) was purchased from Promega (Madison, WI, USA) and β-actin (S-A5441) was purchased from Sigma; RNase A was purchased from Invitrogen (Carlsbad, CA, USA); Propidium iodide was purchased from BD Pharmingen (San Diego, CA, USA).

### Fractionation of HCS

To obtain the water and lipid soluble fractions, HCS was subjected to a solid phase extraction using Sep-Pak C18 Cartridge (Waters Corp., Milford, MA, USA). Briefly, an aliquot of HCS (1 ml) was applied to a preconditioned Sep-Pak solid phase extraction cartridge (1 ml/50 mg, Waters Corp.). The eluate was collected and defined as water soluble fraction. The column was then washed with 1 ml of water and lipid soluble compounds were eluted with 1 ml of ethyl acetate, after which the effluent was collected (lipid soluble fraction). The water-soluble fraction was used as is and the lipid fraction was dried down in a stream of liquid nitrogen and reconstituted to an equivalent volume of DMSO (1 ml) as that of the water soluble fraction.

### Cell culture

Human Burkitt’s B-cell lymphoma cells, Raji, Ramos and Namalwa cells were purchased from American Tissue and Culture Collection (ATCC, Manassas, VA, USA). Mantle cell lymphoma cell line, SP53, was kindly provided by Dr James You at The University of Texas MD Anderson Cancer Center. All cells were cultured in RPMI-1640 medium supplemented with 10% fetal bovine serum (FBS; Hyclone, Logan, UT, USA), 1 mM L-glutamine, and 50 IU/ml penicillin and 50 μg/ml streptomycin. Human peripheral blood mononuclear cells (PBMCs) were purchased from Astarte Biologics (Redmond, WA, USA) and cultured in RPMI-1640 medium. Cells were cultured at 37°C with 5% CO_2_ in a humid atmosphere.

### Cell viability and proliferation

The effect of HCS or bufalin on the growth of lymphoma cells was assessed by the PrestoBlue assay. Raji, Ramos, Namalwa and SP53 cells were seeded at a density of 1×10^4^ cells per well in 96-well plates in RPMI medium and incubated for 24 h. Following incubation, media was replaced and the cells were treated with the indicated concentrations of HCS (0.195–50 μl/ml) or different fraction of HCS, i.e., the water or lipid soluble fractions, or bufalin (1.95–1,000 nM) for 72 h. The cell viability was then measured using the PrestoBlue reagent according to the manufacturer’s instructions. Briefly, 20 μl of the PrestoBlue reagent was added to each well containing 200 μl media. After 1 h of incubation at 37°C, the absorbance was read at a wavelength of 590 nm (Ex/Em, 560/590 nm) using a V-Max Micro-plate Reader by Molecular Devices, Inc. (Sunnyvale, CA, USA). Experiments were repeated at least three times.

### Cell cycle, apoptosis and cell-death

For cell cycle analysis, Ramos cells (2.5×10^6^) grown in 100-mm dishes were treated with HCS (5 and 25 μl/ml) or bufalin (10 and 50 nM) for 24 h. Cells were centrifuged, the pellets were resuspended and washed in 1× PBS, and fixed overnight in 70% ethanol at 4°C. They were then washed with 1× PBS and resuspended in staining solution (PBTB) containing PBS, 0.5% BSA, 0.005% Tween-20, 10 μg/ml propidium iodide (PI) and 1 μg/ml of DNase-free RNase. Cells were incubated in the dark for 30 min at 37°C prior to analysis by fluorescence-activated cell-sorting analysis (FACS) using a FACSCalibur flow cytometer (Becton-Dickinson). The percentage of cells in each phase of the cell cycle was estimated from the DNA histogram content. Apoptotic cell death was further measured by Annexin V surface staining. Briefly, cells (2.5×10^6^) were double stained with fluorescein isothiocyanate (FITC) conjugated Annexin V and PI according to the manufacturer’s instructions (BD Biosciences, San Diego, CA, USA). Fluorescence was detected by the FACSCalibur flow cytometer and analyzed using CellQuest software program (Becton-Dickinson). To determine the effect of caspase-3 inhibitor on HCS induced apoptosis, cells were treated with HCS in the presence or absence of the caspase-3 inhibitor, benzyloxycarbonyl-Asp-Glu-Val-Asp-fluoromethylketone (ZDEVD-fmk) (20 μM) followed by Annexin V staining. For DNA laddering, the DNA was extracted, resolved on a 1% agarose gel, photographed using the Chemidoc-Transilluminator XRS instrument (Bio-Rad, Hercules, CA, USA) and the image captured using the Quantity One software system.

### Immunoblotting

Cytosolic extracts were prepared from Ramos cells treated with HCS with and without ZDEVD as well as bufalin (10 and 50 nM) for 24 h. Briefly, cells were washed in PBS and then resuspended in 50 μl of lysis buffer [20 mM HEPES (*N*-2-hydroxyethylpiperazine-*N*′-2-ethanesulfonic acid), pH 7.5, 10 mM KCl, 1.5 mM MgCl_2_, 1 mM EDTA (ethylenediaminetetraacetic acid), and 1 mM dithiothreitol (DTT)]. After sonication on ice for 3 min with a sonicator 3000 (Misonex Inc., Farmingdale, NY, USA), the protein concentrations were determined by the Bradford assay. Immunoblot assays were performed as per standard procedure. Briefly, equal amounts (50 μg) of protein were subjected to sodium dodecyl sulfate-polyacrylamide gel electrophoresis (SDS-PAGE) followed by transfer to PVDF membranes. Membranes were probed with the indicated antibodies. Secondary antibodies consisting of horseradish peroxidase (HRP)-conjugated goat anti-mouse IgG and anti-rabbit IgG (1:500 vol/vol) were purchased from Santa Cruz. Detection was performed by the enhanced chemiluminescence method from GE Healthcare (Little Chalfont, Buckinghamshire, UK).

### Transmission electron microscopy

Ramos cells (5×10^6^) were seeded in 100-mm dishes. The cells were then incubated at 37°C, 5% CO_2_ for ~12–24 h. Following treatment with HCS (0, 25 μl/ml) for 24 h, the cells were harvested by centrifugation at 3,000 rpm for 2 min. After two washes with 1× PBS, the pellet was resuspended in fixative (2% paraformaldehyde and 3% gluteraldehyde) and stored at 4°C. Samples were fixed with a solution containing 3% glutaraldehyde plus 2% paraformaldehyde in 0.1 M cacodylate buffer, pH 7.3, for 1 h. After fixation, the samples were washed and treated with 0.1% Millipore-filtered cacodylate buffered tannic acid, postfixed with 1% buffered osmium tetroxide for 30 min, and stained en bloc with 1% Millipore-filtered uranyl acetate. The samples were dehydrated in increasing concentrations of ethanol, infiltrated, and embedded in LX-112 medium. The samples were polymerized in a 60°C oven for 2 days. Ultrathin sections were cut in a Leica Ultracut microtome (Leica, Deerfield, IL, USA), stained with uranyl acetate and lead citrate in a Leica EM Stainer, and examined in a JEM 1010 transmission electron microscope (Jeol, USA, Inc., Peabody, MA, USA) at an accelerating voltage of 80 kV. Digital images were obtained using AMT Imaging System (Advanced Microscopy Techniques Corp., Danvers, MA, USA).

### Gene expression

Ramos cells (2.5×10^6^) were seeded overnight in 100-mm dishes and were then treated with 25 μl/ml of HCS for 24 h. Total RNA was extracted using an RNeasy kit (Qiagen, Valencia, CA, USA). Gene expression analysis was performed at the The University of Texas MD Anderson Sequence and Microarray Core facility, using the Affymetrix Gene chip 1.0 ST. Gene expression analysis was normalized by β-actin expression and set to 1 for the control DMSO-treated cells.

### Statistical analysis

Student’s t-test was used to determine the statistical differences between various experimental groups; a value of P≤0.05 was considered statistically significant.

## Results

### HCS and its lipid fraction inhibit proliferation of lymphoma cell lines but not human PBMCs

**Figure 1:**
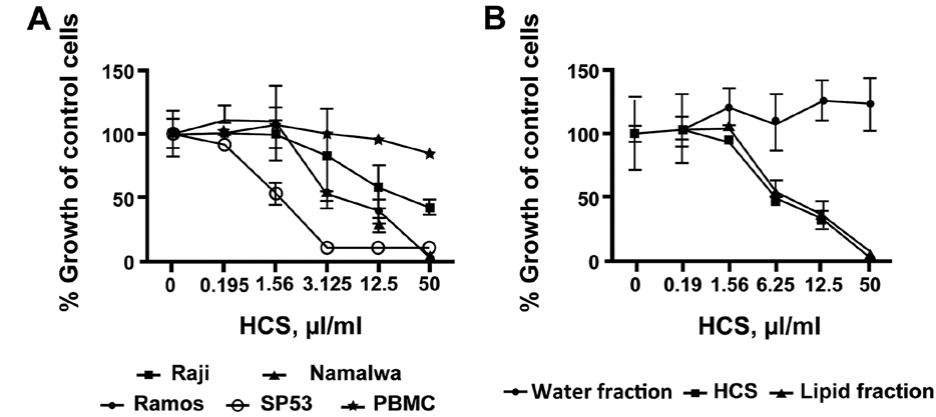
Huachansu (HCS) inhibits cell proliferation of human non-Hogdkins lymphoma cells. (A) Burkitt’s lymphoma cells Raji, Ramos, Namalwa, mantle cell lymphoma (SP53), and normal PBMC cells were treated with HCS (0.02–50 μl/ml) for 72 h. (B) Ramos cells were treated for 72 h with HCS, water and lipid fraction at the indicated concentrations. Cell proliferation was evaluated by the PrestoBlue assay. Each experiment was performed in quadruplicate and repeated at least twice independently. Data are presented as mean ± SD.

To investigate the effects of HCS on cell proliferation in lymphomas, we treated three human Burkitt’s non-Hodgkin’s lymphoma cell lines, Raji, Ramos and Namalwa as well as mantle cell lymphoma SP53 cells with various concentrations of HCS. HCS markedly inhibited cell proliferation in all three cell lines tested in a dose- and time-dependent manner, with IC_50_ of inhibition ranging from 3.125 μl/ml for Ramos and Namalwa to 25 μl/ml for Raji cells (Fig. 1A). A comparable level of antiproliferative effect, IC_50_ of ~1.5 μl/ml, was achieved when mantle cell lymphoma SP53 cells were treated with HCS (Fig. 1A). In contrast to the lymphoma cells, HCS did not affect the proliferation of human PBMC (Fig. 1A). Because a number of bioactive components either water or lipid soluble are present in HCS, we tested for antiproliferation of the aqueous and lipid fractions in the Ramos cells. In Fig. 1B, the lipid fraction exhibited antiproliferative activity comparable to the parent HCS whereas the aqueous fraction showed no antiproliferative activity in these cells, suggesting that the lipid fraction is responsible for the antiproliferative effect in HCS.

### Effects of HCS on the cell cycle and cell death in Ramos cells

**Figure 2:**
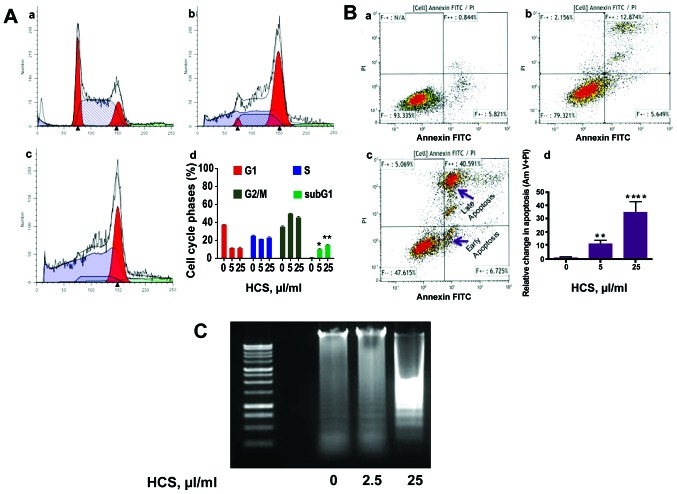
HCS triggers cell arrest and induces cell death of Ramos cells. Cells were treated with HCS for 24 h. (A) Histograms of Ramos cells treated with vehicle (a), HCS 5 μl/ml (b) and HCS 25 μl/ml (c) and quantitative analysis of the cell cycle alteration elicited by HCS (d). (B) Cells were treated with HCS at similar concentrations illustrated in sub-figure A, and apoptotic cells were assayed by flow cytometry following Annexin V staining, using a FACSCalibur instrument. Each experiment was performed in duplicate and repeated twice independently. Each bar graph represents the mean and the error bars represent ± SEM. ^*^P<0.05, ^**^P<0.01, ^****^P<0.001 statistically significant difference between HCS treated versus vehicle treated cells. (C) Ramos cells were treated with HCS at 2.5 and 25 μl/ml for 24 h. The DNA was extracted, resolved on a 1% agarose gel, stained with ethidium bromide and photographed.

We next investigated the type of cell death elicited by HCS in Ramos cells, which is most sensitive to HCS treatment. After treatment with increasing concentrations (0, 5 and 25 μl/ml) of HCS for 24 h, cells were subjected by Annexin V/propidium iodide (PI) staining followed by flow cytometry analysis. HCS treatment resulted in a dose-dependent increase in sub-G1 phase arrest (Fig. 2A-b and -c) compared to vehicle treated cells (Fig. 2A-a). Compared to control cells, there was a ~10-fold increase in sub-G1 phase cells in HCS (25 μl/ml) treated samples, suggesting the induction of apoptosis (Fig. 2A-d). The induction of apoptosis by HCS in Ramos cells was further evidenced by increased both early and late apoptotic cell population with Annexin V- and PI staining, in a dose-dependent manner (Fig. 2B-b and c) compared to control cells (Fig. 2B-a). In fact, HCS 25 μl/ml increased late stage apoptosis by almost 35-fold compared to vehicle treated cells (Fig. 2B-d). The formation of internucleosomal DNA fragments is frequently used as a marker for cells undergoing programmed cell death. Fig. 2C is indicative of the DNA laddering pattern triggered, in a dose-dependent manner, by HCS treatment.

### HCS modulates key regulatory proteins critical for cell growth in lymphoma cells

**Figure 3:**
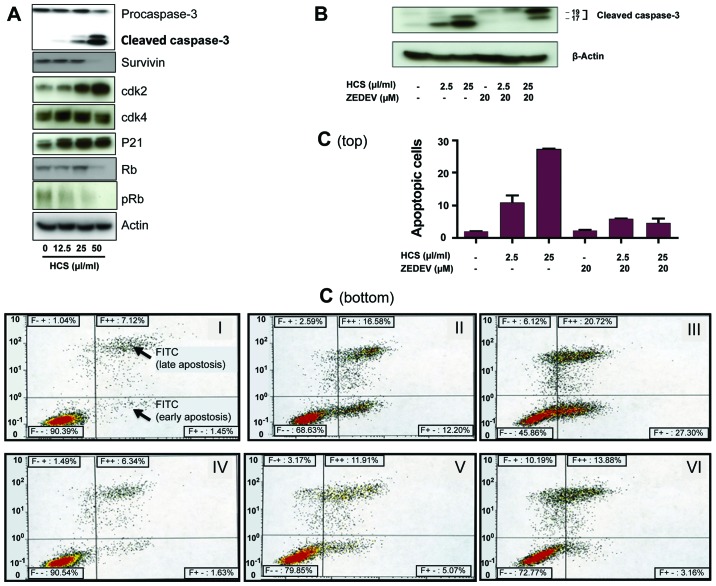
Modulation of apoptotic and cell cycle regulatory proteins in Ramos cells treated with HCS. (A) Ramos cells were treated with HCS (0, 12.5, 25 and 50 μl/ml) for 24 h, followed by western blot analysis to detect caspase-3, cdk2, cdk4, p21^CIP1^, survivin, Rb and pRb proteins. (B) Cells were treated with the indicated concentrations of HCS with or without the caspase inhibitor, ZDEVD, for 24 h and immunoblotted with the caspase-3 antibodies. (C) The effect of ZDEVD on HCS induced apoptotic cell death by Annexin V staining. ZDEVD markedly blocked the early phase of apoptosis induced by HCS while moderately modulated the late phase of apoptotic or necrotic cells (top, bar graph of early phase apoptosis; bottom, histogram). Each experiment was performed in duplicate and repeated twice independently. Data are presented as mean ± SD.

We next investigated how cell cycle and apoptotic cell death regulatory proteins were impacted by HCS. Immunoblotting analyses showed an increase of the cyclin-dependent kinase inhibitor p21^CIP1^, a key regulator of the cell cycle, at a low concentration of 12.5 μl/ml. In contrast, there was a dose-dependent decrease in phosphorylated Rb (pRb) protein and survivin (Fig. 3A). Additionally, HCS resulted in a dose-dependent increase of caspase-3, as this caspase is cleaved from the procaspase to an active caspase when cells undergo caspase-dependent apoptotic cell death. To further confirm the role of caspase-3 in HCS-induced cell death, Ramos cells were treated with HCS and the caspase-3 specific inhibitor ZDEVD-FMK for 24 h, followed by detection of active caspase-3 by western blot analysis. Fig. 3B shows that HCS alone induced formation of active caspase-3, in a dose-dependent manner, whereas ZDEVD alone inhibited active caspase-3 formation. The combination of HCS and ZDEVD abrogated the HCS effect alone, as evidenced by the marked decrease in the 17-kDa fragment of caspase-3 in the ZDEVD. In line with this, the results of Annexin V staining of Ramos cells treated with ZDEVD alone and in combination with HCS mirrored the active caspase-3 cell death depicted in Fig. 3B, as the combination of ZDEVD and HCS significantly reduced the cells undergoing the early phase apoptosis and partially abrogated late phase apoptotic cell death (Fig. 3C, V and VI) compared to that caused by HCS alone (Fig. 3C, II and III), whereas ZDEVD alone did not affect cell death (Fig. 3C, IV) compared to that of control group (Fig. 3C, I).

### Ultrastructural changes of Ramos cells by transmission electron microscopy

**Figure 4:**
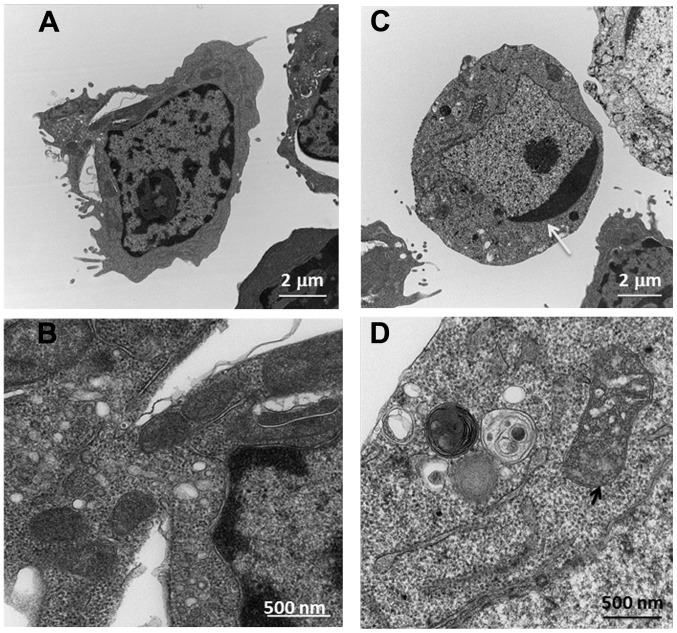
Ultrastructure of Ramos cells treated with HCS by transmission electron microscopy. Ramos cells were treated with HCS (0 and 25 μl/ml) for 24 h. Following treatment, cells were harvested and processed for TEM, as described in Materials and methods. (A) Control Ramos cells (x5,000). (B) Control Ramos cells (x10,000); (C) HSC (25 μl/ml) treated Ramos cells (x5,000); (D) HSC (25 μl/ml) treated Ramos cells (x10,000). The white arrow indicates condensed chromosome in HCS treated Ramos cells, while the black arrow points to enlarged and swollen mitochondria in the HCS treated Ramos cells.

To further confirm that HCS mediated cell death through apoptosis in Ramos cells, we determined the ultrastructural changes of Ramos cells after being treated with HCS using transmission electron microscopy (TEM). As shown in Fig. 4, HCS (25 μl/ml) induced marked ultra-structural changes in cellular organelles, especially condensed chromatin in the nucleus and distorted mitochondria (see arrows in Fig. 4C and D) compared to that of control treated cells (Fig. 4A and B), further suggesting that the antiproliferative effect of HCS is being mediated through the induction of apoptosis.

### HCS affects MAP kinase gene and protein expression

**Figure 5:**
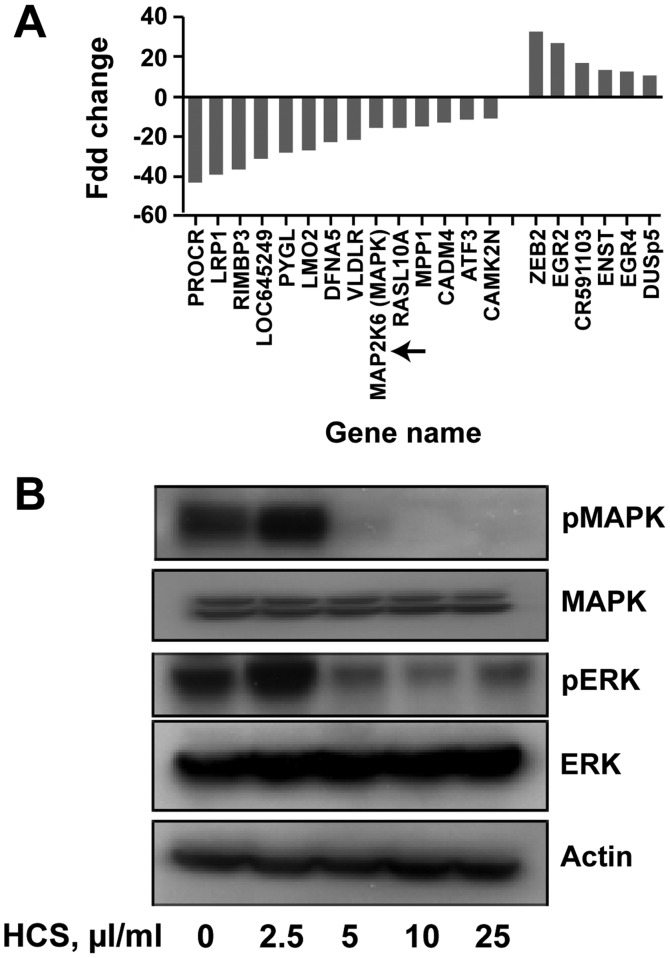
Gene expression analysis and assessment of signaling pathways perturbed by HCS in Ramos cells. Cells were treated with 25 μl/ml of HCS for 24 h and gene expression analysis was performed at the MD Anderson microarray core facility, using an Affymetrix chip. (A) Genes were up- and downregulated ~10–40-fold in HCS treated Ramos cells relative to vehicle treated cells. (B) The expression of MAP kinase pathway proteins in Ramos cells treated with HCS at the indicated concentrations for 24 h. Cell lysates were then subjected to western blot analysis for total and phosphorylated levels of MAPK and ERK proteins.

To determine the molecular mechanisms associated with HCS-induced cell death on Ramos cells at the transcriptional level, we treated Ramos cells with 25 μl/ml of HCS for 24 h and the alteration of gene expression was assessed using an Affymetrix chip. All genes showing 10–40-fold up- or down-regulation are summarized in Fig. 5A. Among these highly regulated genes, the mRNA expression of MAP2K6 was reduced by 19-fold compared to that of vehicle treated Ramos cells. To gain further insights into whether HCS also regulated the MPA kinase translationally, we treated Ramos cells with HCS for 24 h and determined the protein expression of MAP kinase by immunoblotting. As shown in Fig. 5B, expression of phosphorylated MAPK and ERK was suppressed in a concentration-dependent manner, with no changes in the total MAPK and ERK, potentially implicating HCS in the inhibition of the MAP kinase signaling pathway. Together, these findings suggest that, in addition to the caspase-3-mediated cell death, HCS might also induce cell death in lymphomas through inhibition of MAP kinase signaling.

### Cardiac glycosides, the major component of HCS, inhibited Ramos cells by induction of apoptosis

**Figure 6:**
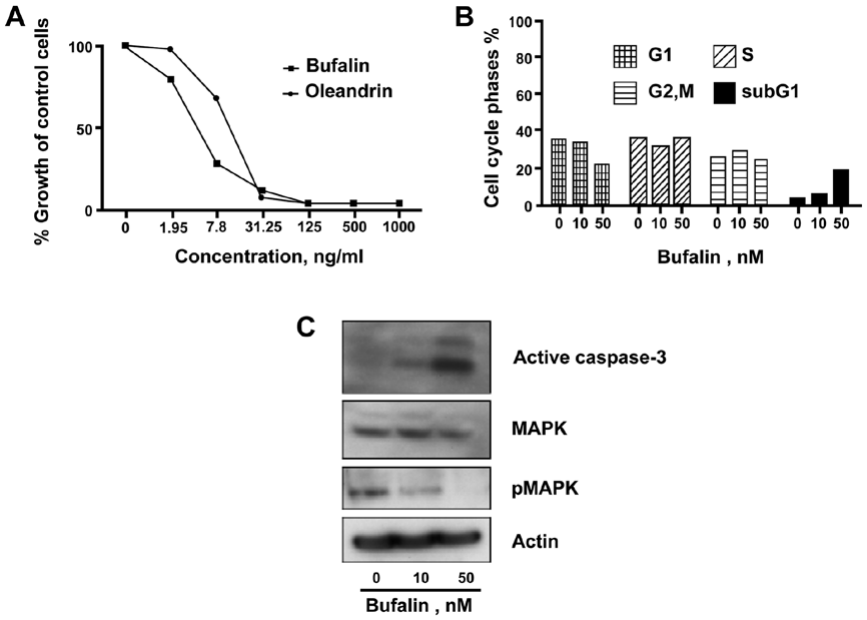
Effects of bufalin on proliferation, cell cycle and cell signaling proteins in Ramos cells. (A) The effect of bufalin and oleandrin in the growth of Ramos cells that were treated with these particular agents for 72 h. Proliferation assay using the PrestoBlue reagent was performed as described in Materials and methods. The unit of conversion for bufalin is 10 ng/ml = 26 nM and for oleandrin, 10 ng/ml = 17 nM. (B) Cell cycle and apoptotic cell death in Ramos cells treated with bufalin (10 and 50 nM) for 24 h. (C) The expression of active caspase-3 and MAP kinase in Ramos cells treated with bufalin for 24 h.

Bufalin, the major cardiac glycoside present in HCS, has been shown to have relatively potent anticancer activity in various solid tumors (24). To test its antiproliferative effect in NHL, we treated Ramos cells with bufalin and another cardiac glycoside, oleandrin, for 24 and 72 h and assayed for cell cycle and proliferation, respectively. The proliferation analysis showed a marked growth inhibition of Ramos cells with an IC_50_ of 5±0.15 ng/ml and 15±0.18 ng/ml for bufalin and oleandrin, respectively (Fig. 6A). The cell cycle analysis showed a dose-dependent increase in the sub-G1 cell population, following treatment of Ramos cells with bufalin at a relatively low concentration of 50 nM (Fig. 6B). Similar to the effect of HCS, bufalin treatment of Ramos cells increased formation of activated caspase-3 as well as decreased pMAPK levels (Fig. 6C).

## Discussion

Nature abounds with drugs that are exploited by humans for the treatment of a variety of ailments (25,26). Many anti-cancer agents are derived from natural sources, primarily from plants (27). Animal-derived anticancer drugs are also available, including ARA-C, modeled after compounds from the Caribbean sponge, used to treat leukemia and lymphoma (28). The drug TM 601 is derived from the Israeli yellow scorpion and attacks malignant glioma tumors, without harming healthy cells (29). ET 743, which comes from sea squirts, is being tested for treatment of ovarian cancer and soft tissue sarcoma (30–32). A number of marine natural products and related compounds have progressed on to clinical trials (33). Herein, we report that HCS inhibited cell proliferation, and induced cell death through apoptosis in human NHL cells.

In these investigations, we found that HCS, at clinically achievable doses, significantly inhibited the proliferation of a number of NHL cells, especially Ramos. The antiproliferative effect of HCS in NHL appears to be mediated through induction of apoptosis of Ramos cells via activation of caspase-3 pathway. Intriguingly, HCS also downregulated the MAP kinase translationally and transcriptionally in Ramos cells. Given that the MAP kinase pathway has been recognized as one of the most important oncogenic pathways in aggressive B-cell lymphoma (34,35), HCS certainly warrants further investigation.

**Figure 7:**
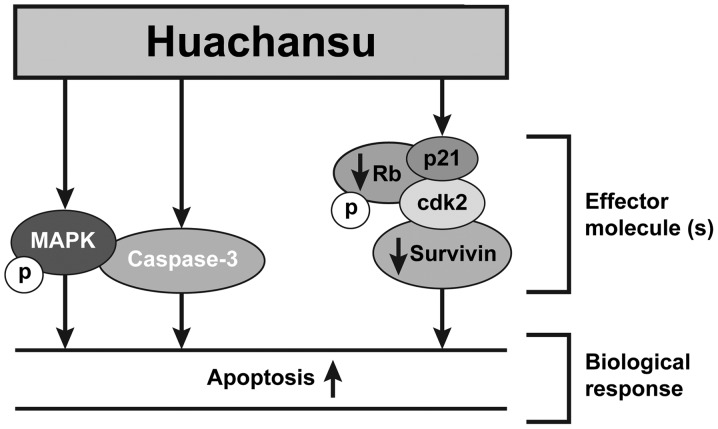
Proposed signaling pathway modulation in lymphomas by HCS.

As a hot water extract of toad skin, HCS contains multiple biologically active components. To ascertain the most active components that are responsible for HCS-mediated anticancer activity, we separated HCS into lipid- or water-soluble components using reverse phase solid phase extraction method. As depicted in Fig. 1B, the lipid component inhibited proliferation comparable to the HCS mixture, whereas the water-soluble fraction had no measurable effect. These results indicated that the lipid-fraction of HCS is primarily responsible for its antiproliferative activity. We, and others, have reported that cardiac glycosides (bufadeinolides), especially, bufalin, the major bioactive component of HCS are responsible for HCS-mediated anticancer activity in various solid tumors (36). However, whether bufalin is also capable of inhibiting the proliferation of NHL, particular Burkitt’s B-cell lymphoma, is lacking. In this study, our results showed that, at as low as 5 ng/ml, bufalin inhibited proliferation of Ramos cells by 50% suggesting that it has potent anticancer effect in NHL cells. Additionally, bufalin inhibited proliferation of Ramos cells through induction of apoptosis, activation of the caspase-3 pathways, and inhibition of the MAP kinase pathway (Fig. 7, model), suggesting that bufalin could be a main bioactive component responsible for HCS anticancer activity in aggressive B-cell lymphomas.

The proapoptotic effect of HCS or its bioactive components, such as cardiac glycosides, has been reported in solid tumor derived cell lines, such as hepatocellular carcinoma HepG2 cells, and proposed to act mainly through downregulation of mitochondria- and Fas-mediated caspase-dependent pathway (6). Active caspase-3 is a homodimer of heterodimers and is produced by proteolysis of procaspase-3 (37). Programmed cell death (apoptosis) can occur through caspase-dependent and -independent pathways (38). In light of the previous study, we reasoned that HCS might also directly activate procas-pase-3 and cause induction of apoptosis in B-cell lymphoma cells. Indeed, when Ramos cells were treated with HCS with and without the caspase inhibitor, ZDEVD, a cell-permeable, irreversible inhibitor of caspase-3/CPP32 that is known to inhibit cell apoptosis, ZDEVD blocked formation of lower molecular forms of active caspase-3 induced by HCS. In line with this, ZDEVD also partially blocked the proapoptotic activity of HCS, suggesting the activation of caspase-3 could be in part attributable to HCS antiproliferative effect in Ramos cells. This is consistent with previous research with HCC cells (6). In HCC cell lines treated with HCS, the activation of caspase-9 was also observed (6,39). In contrast, the expression of caspase-9 in the HCS treated Ramos cells was not altered (data not shown), suggesting the HCS-induced apoptotic effect is mediated through different molecular mechanisms in HCC and B-cell lymphomas. More detailed biochemical *in vitro* assays in cell lines and in freshly isolated tumors as well as *in vivo* tumor regression analyses in animal models need to be conducted to gain a better idea of the mode of action of HCS.

In contrast to caspase-3 activation by HCS, MAP kinases gene and protein were downregulated in Ramos cells treated with HCS. MAPKs are widely expressed serine-threonine kinases that mediate important regulatory signals in the cell. Activation of MAPK pathway is a critical event for a number of solid tumors as well as NHL (40). For example, Green *et al* reported that the genetic alteration of the MAPK and apoptotic pathways alone or with genetic amplification of FOXM1 as a conserved mechanism of lyphomagenesis in NHL including FLs, DLBCLs and B-CLL (41). Three major MAP kinase pathways, designated by their terminal kinases, have been extensively studied: the extracellular signal-regulated kinase (ERK1/2), c-Jun N-terminal kinase (JNK1/2), and p38 kinase pathways. These MAP kinases are activated via a series of sequential phosphorylations of upstream kinases, and they function primarily to transduce signals to the cell nucleus, ultimately affecting gene expression. The role of bufalin on MAP kinase has been studied by a number of investigators suggesting that bufalin induces apoptosis by activation of MAPKK1 and JNK pathways in human leukemia U937 and HL-60 cells. In contrast, Jiang *et al* reported that bufalin inhibited the phosphorylation of Akt, NF-κB, p44/42 MAPK (ERK1/2), and p38 MAPK in A549 cells (42) suggesting MAP kinase could be differentially regulated by bufalin depending on tumor cell types.

The current study is the first to examine the effect of HCS on MAPK pathways in relation to its induced cell death in lymphomas, especially NHL. We showed that HSC at as low as 5 μl/ml blocked almost 90% phosphorylation of MAPK and 50% phosphorylation of ERK while no changes were observed with total MAPK expression. Similarly, bufalin also decreased MAPK phosphorylation in a dose-dependent manner in Ramos cells. Taken together, our data suggest that HCS mediates cell death possibly through modulation of the MAP kinase pathway.

In conclusion, we reported that HCS can potently inhibit proliferation of NHL, especially Burkett’s non-Hodgkin’s lymphoma cells. The anticancer activity of HCS appears to be linked to the induction of apoptosis in non-Hodgkin’s lymphomas by specific activation of caspase-3. Additionally, MAP kinases were also notably downregulated. This is the first study suggesting the anticancer potential of HCS in hematologic malignancies. Given that the result of our phase I study on HCS and solid tumors has suggested that HCS is well tolerated with minimum side effects at doses as high as 120 ml/m^2^, which is almost 6-fold higher than the dose (20 ml/m^2^) regularly used in oncology clinics in China, HCS, therefore, warrants further investigation as a novel treatment modality in NHL.

## Abbreviations

HCS: Huachansu
NHL: non-Hodgkin’s lymphoma
BL: Burkitt’s lymphoma
MCL: mantle cell lymphoma
TCM: traditional Chinese medicine
VEGF: vascular endothelial growth factor
EGFR: epidermal growth factor receptor
MAPK: mitogen-activated protein kinase
ZDEVD: benzyloxycarbonyl-Asp-Glu-Val-Asp-fluoromethylketone
DMSO: dimethyl sulfoxide
PI: propidium iodide
PBMC: peripheral blood mononuclear cell
FBS: fetal bovine serum
FACS: fluorescence-activated cell-sorting analysis
FITC: fluorescein isothiocyanate
HEPES: *N*-2-hydroxyethylpiperazine-*N*′-2-ethanesulfonic acid
EDTA: ethylenediaminetetraacetic acid
SDS-PAGE: sodium dodecyl sulfate-polyacrylamide gel electrophoresis
TEM: transmission electron microscopy
